# Integrated Ecosystem Assessments: Developing the Scientific Basis for Ecosystem-Based Management of the Ocean

**DOI:** 10.1371/journal.pbio.1000014

**Published:** 2009-01-20

**Authors:** Phillip S Levin, Michael J Fogarty, Steven A Murawski, David Fluharty

## Abstract

Integrated ecosystem assessments challenge the broader scientific community to move beyond the important task of tallying insults to marine ecosystems to developing quantitative tools that can support the decisions national and regional resource managers must make.

A series of prominent and controversial papers about the state of marine ecosystems has occupied the pages of high-profile journals over the last decade [1–7]. While some might quarrel with the specific conclusions of these papers, there is no dispute that managers of ocean and coastal habitats confront a growing diversity of very serious challenges [8] that, if left unattended, threaten the ability of marine ecosystems to supply the goods and services required or desired by humans [9].

The tenets of ecosystem-based management (EBM) now occupy center stage in our efforts to rebuild marine ecosystems. Indeed, over the last several decades EBM has evolved from a vague principle to a central paradigm underlying living marine resource policy in the United States [10,11]. EBM differs from conventional resource management in that it defines management strategies for entire systems, not simply individual components of the ecosystem [12]. As a consequence, EBM takes into account interactions among ecosystem components and management sectors, as well as cumulative impacts of a wide spectrum of ocean-use sectors [13]. Importantly, EBM considers humans as an integral part of the ecosystem, since humans derive a portfolio of services from the ecosystem and also act as a driver influencing ecosystem processes. Thus, a key aspect of EBM is illuminating trade-offs among ecosystem services and management goals [14]. After years of debating about the meaning of EBM, and whether EBM is possible or even needed, we have arrived at a turning point where large-scale, comprehensive EBM is broadly accepted as crucial for effective marine conservation and resource management [15].

While some policy makers clearly grasp the utility of an EBM approach, implementation of EBM in marine ecosystems is a significant hurdle, and little practical advice is available to inform management authorities on how to select specific management measures to achieve EBM goals. Here we propose “integrated ecosystem assessments” (IEAs) as a framework for organizing science in order to inform decisions in marine EBM at multiple scales and across sectors. Below we describe our view of IEAs, highlighting the ways that they will enhance the ability of resource managers to evaluate cumulative impacts of diverse human activities as well as steer management efforts to achieve multiple simultaneous ecosystem objectives. The approach we outline follows the paradigm of formal decision analysis [16], is consistent with the Millennium Ecosystem Assessment [9], and is a descendant of approaches advocated by Caddy [17], Sainsbury [18], and Smith [19]. While developed with marine ecosystems in mind, the IEA framework aims to guide the process of synthesizing and analyzing relevant scientific information supporting an ecosystem approach in any system.

We define an IEA as a formal synthesis and quantitative analysis of information on relevant natural and socioeconomic factors, in relation to specified ecosystem management objectives. It is an incremental approach, in which integrated scientific understanding feeds into management choices and receives feedback from changing ecosystem objectives. This approach involves and informs citizens, stakeholders, scientists, resource managers, and policy makers through formal processes that contribute to attaining the goals of EBM. IEAs, as we envision them, do not necessarily supplant single-sector management; instead, they inform the management of diverse, potentially conflicting ocean-use sectors. As such, we view IEAs as a necessary supplement to, and extension of, single-species and single-sector approaches.

## A Five-Step Process for IEAs

Below we outline five key steps that, we contend, are necessary for IEAs and that enhance the likelihood of successful implementation of EBM. These are scoping, indicator development, risk analysis, management strategy evaluation, and ecosystem assessment (Figure 1).

**Figure 1:**
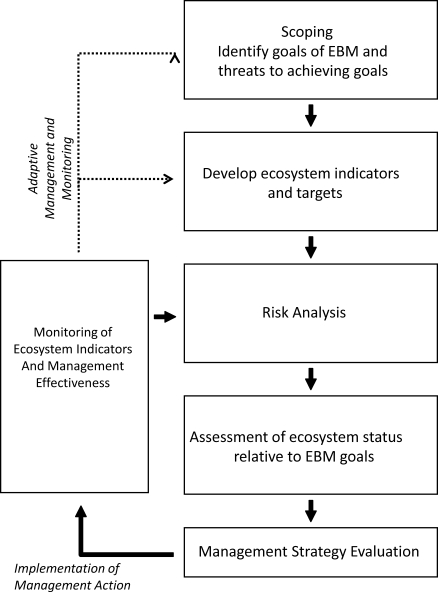
The Five-Step Process of Integrated Ecosystem Assessment An IEA begins with a scoping process to identify key management objectives and constraints, identifies appropriate indicators and management thresholds, determines the risk that indicators will fall below management targets, and combines risk assessments of individual indicators into a determination of overall ecosystem status. The potential of different management strategies to alter ecosystem status is evaluated, and then management actions are implemented and their effectiveness monitored. The cycle is repeated in an adaptive manner.

### Scoping.

The IEA process begins with a scoping step. It is in this step that specific ecosystem objectives and threats are identified. While EBM is, by definition, more inclusive than traditional sectoral approaches, IEAs cannot evaluate all issues relevant to a specific ecosystem milieu because of limitations in understanding the totality of ecosystem interactions. Thus, this first step of the IEA results in an abstraction of ecosystems into sub-systems thought to be most influential to the management issues at hand. Scoping involves identification of critical ecosystem management drivers and specific pressures on ecosystems. It must then place issues in a broad systems context, investigate stakeholder interests and agendas, and identify patterns of interaction among stakeholders [20].

Importantly, scoping is a process in which stakeholders are deeply involved. Stakeholder participation is particularly relevant in marine systems because issues and interests cross ecological, social, and political boundaries; are subject to multiple uses, users, and objectives; often have unclear or open-access property rights; and contain multiple ecosystem services, which are not traded competitively and have no monetary value in the marketplace [21].

Gregory and Wellman [22] discuss a comprehensive scoping process for Tillamook Bay, Oregon. The Tillamook Bay watershed covers over 59,488 hectares and supports numerous species of both economic and cultural importance, including shellfish, salmon, trout, and numerous bottom fish. The watershed is also home to numerous forestry and agricultural activities (especially dairy farming) that contribute to the regional economy but also have the potential to impact the bay. The Tillamook Bay National Estuary Project was charged with identifying and evaluating management actions that could restore degraded aspects of the Tillamook Bay ecosystem. To whittle down an initial list of about 150 proposed management actions, technical experts, community leaders, and stakeholders participated in a scoping process that included carefully crafted questions and small-group discussions with a local facilitator. Through this process, the group was able to refine their objectives and deconstruct problems and actions into their constituent parts. As a consequence, three crucial, though controversial, actions (limiting livestock access to streams, protecting and restoring tidal wetlands, and upgrading forest management roads) were highlighted for subsequent consideration.

Similarly, intensive scoping and stakeholder involvement have been critical to the development of ecosystem-based fishery management in a number of regions in the US. For instance, in 2005, facilitated workshops were held in 21 coastal communities from North Carolina to Maine. These workshops used a structured questionnaire and open discussions in order to gauge stakeholder opinions on various traditional and ecosystem-based fishery management actions. The stakeholder views garnered from this process are now explicitly being used by the National Marine Fisheries Service to identify management options and will be used to quantitatively evaluate various management scenarios.

Although often underemphasized, the scoping process frequently determines the success or failure of an IEA (see [23]), as well as greatly influencing the decision environment in which policies are considered [24]. Importantly, the scoping step assumes a governance system capable of organizing discussions relevant to IEAs. However, ocean governance can be fragmented with spatial and temporal mismatches between the scales of governance and the ecosystem [25]. Examples of appropriate governance structures do exist (e.g., [26]), and these are critical because in their absence scientists are left to debate the causes and consequences of ecosystem-level impacts without an appropriate management authority to inform or a mechanism to effect needed changes.

### Indicator development.

Following the scoping process, appropriate indicators of ecosystem state must be identified and validated (Figure 1). Carefully selected indicators provide the basis for the assessment of status and trends in ecosystem state. In some cases, indicators will simply track the abundance of a single species (as in the case of an endangered species). More often, indicators will serve as proxies for ecosystem attributes of interest (e.g., resistance to change, resilience to perturbation, or maintenance of critical service functions). Resiliency to perturbation, for example, is an attribute of interest [27], and species diversity may be one indicator of ecosystem resiliency. In situations that are data-rich, a key consideration is to avoid compilation of numerous uninformative time series in favor of a few synthetic and responsive indicators of ecosystem state. Ideally, historical measurements of selected indicators inform the establishment of target and threshold values of quantities relevant to management (e.g., maximum nutrient inputs, minimum stock size limits for fisheries and endangered species conservation). Suites of indicators should be chosen that span a wide range of processes (with different associated rates), biological groups, and indicator types (“tactical” and “strategic,” “early warning,” and “integrated system state”) [28].

There is no dearth of potential indicators of ecosystem status, but the real work is to wisely select from among a long list of potential indicators [29,30]. Rice and Rochet [31] outline a useful framework for identifying a suite of informative indicators for EBM. They argue that indicators should be directly observable and based on well-defined theory, while also being understandable to the general public, cost-effective to measure, supported by historical time series, sensitive and responsive to changes in ecosystem state (and management efforts), and responsive to properties they are intended to measure. The Rice and Rochet process provides a formal method for assigning weights to each of these criteria, which can then be used in collaboration with managers to select the final suite of indicators.

Computer simulation provides a powerful approach for evaluating indicator performance. For instance, Fulton [28] used the Atlantis ecosystem model to determine the degree to which potential indicators reflect changes in ecosystem attributes. In this approach, an operating model is used to simulate the dynamics of the system over time. A sampling model is used to simulate a monitoring program and produces a time series of pseudo-data with realistic sampling and process error. These pseudo-data are then processed using standard techniques to generate time series of indicators. Indicators are then evaluated by their ability to detect or predict changes in “true” values of key ecosystem attributes (which are known from the simulation model). In regions with limited data or resources, these ecosystem simulations can be used to highlight indicators that are informative and cost-effective to measure.

### Risk analysis.

Once ecosystem indicators are selected, the next IEA step evaluates the risk to the indicators posed by human activities and natural processes. The goal of these risk analyses is to qualitatively or quantitatively determine the probability that an ecosystem indicator will reach or remain in an undesirable state. Ecosystem modeling and analysis are important in determining incremental improvements in ecosystem indicators in response to changes in human-induced pressures. Risk analysis must explicitly consider the inevitable uncertainties involved in understanding and quantifying ecosystem dynamics and their positive and negative impacts on social systems.

A number of analytical techniques might fruitfully be used for ecosystem-scale risk analyses (e.g., [32]). For example, Smith et al. [19] outline a hierarchical approach that employs analytical methods ranging from qualitative through fully quantitative models. Their qualitative method relies on expert opinion to characterize the scale, intensity, and consequence of particular threats that have been identified by stakeholders. Those hazards that present “moderate” or greater risk are then subjected to further analysis. For each species or indicator that warrants further analysis, two scores are derived. The first describes the probability that a species or indicator will be exposed to an impact (i.e., susceptibility), and the second expresses the ability of the species or attribute to recover from impact (i.e., resilience). The overall risk score is derived by integrating these two components of risk. Thus, indicators with low resilience and high susceptibility have high risk, while those indicators with high resilience and low susceptibility have low risk. Again, those indicators with at least moderate risk are subjected to further analysis. In this case, existing quantitative models (e.g., population viability analysis, fisheries stock assessment) could be used to rigorously determine the probability that indicators will cross a management benchmark.

Results from the risk analysis for each ecosystem indicator are then integrated in the assessment phase of the IEA. Using statistical models, the assessment quantifies the status of the ecosystem relative to historical status and prescribed targets. Thus, the risk analysis rigorously quantifies the status of individual ecosystem indicators, while the full assessment considers the state of all indicators simultaneously.

### Management strategy evaluation.

The next phase of the IEA uses ecosystem modeling frameworks to evaluate the potential of different management strategies to influence the status of natural and human system indicators. To accomplish this, a formal management strategy evaluation (MSE) can be employed [18]. In MSE, models are used to simulate the behavior of ecosystems and provide the ability to forecast changes in ecosystem state as a consequence of management scenarios and decision rules. MSE in the context of an IEA can thus serve as a filter to identify which policies and methods have the potential to meet stated objectives.

The MSE approach has been used with great success in a number of fisheries settings (e.g., review by [33]), and while the uncertainty of ecosystem models presents serious challenges, this method holds great promise for ecosystem assessment and management [19]. For example, in Southeast Australia MSE was used very effectively to provide insight into the potential consequences of different fisheries management scenarios on tradeoffs between various ecological and socioeconomic objectives [34]. A key finding of this MSE was that no single management scenario will consistently provide an optimal outcome across all management goals. Trade-offs have to be made, and this approach illuminates what the trade-offs are as well as how to operate along those trade-offs.

### Monitoring and evaluation.

The final stage of the IEA process consists of continued monitoring and assessment of ecosystem indicators. Without ongoing monitoring and evaluation of the effectiveness of management actions, we have no way of knowing if management strategies are working and lack the ability to learn from our failures [35]. While monitoring the effectiveness of management actions seems obvious, such monitoring is costly and frequently poorly done. For example, Rumps and colleagues [36] in a recent review of 23,000 projects noted that following restoration of salmon habitat, more than a third of projects had insufficient monitoring to determine if a management action was successful. Additionally, while more than two thirds of projects reported success, fewer than half the projects had clear criteria for what success meant. Inadequate effectiveness monitoring clearly leads to delays in management response, particularly if management actions involve economic loss [37]. Such delays can result in further degradation of the system, making appropriate management all the more difficult.

## The Importance of Scale

Formal IEAs force decision makers to squarely confront both the spatial and temporal scales over which ecosystem dynamics, management issues, and societal impacts occur. Scales must be consistent with the ability to recognize and explain the most important drivers and threats to the ecosystem. Ecosystems typically do not have sharp boundaries; rather, ecosystems blend into each other, and ecosystem components may overlap or interact at multiple scales (e.g., highly migratory species like tuna traverse and dynamically link adjacent ecoregions). As a consequence, ecosystem boundaries are human constructs, and IEAs must identify a spatial scale in the context of the issues and problems under consideration. The scales over which IEAs apply will naturally have to be flexible, owing to the nature of the issues and the involvement of local, state, regional, and federal management authorities in the management of ecosystems. Integrated assessments must, then, use tools that can imbed hierarchical scales to inform management problems along this continuum. Within this broader spatial context, we envision nested spatial management strategies such as the use of zoned usage patterns. Additionally, IEAs must incorporate appropriate temporal scales. In particular, IEAs require attention to the temporal baseline against which current status is compared. Different conclusions may be drawn, for example, when comparing the current status of ecosystem indicators to those measured 25 years versus 75 years ago [38].

## Applying the IEA Concept

The basic IEA approach is rooted in formal decision theory, and as in other applications of this paradigm, implementation forces practitioners to confront a dizzying array of issues [16]. The approach allows us to quantitatively consider objectively and subjectively identified goals in an open and transparent setting. Identifying and evaluating trade-offs among diverse and possibly incommensurable objectives is feasible within this general setting.

In marine ecosystems, issues span sectors as diverse as fisheries, tourism, energy, shipping, real estate, agriculture, and forestry (among many others). Despite the complexity of the issues, aspects of the IEA framework have been successfully used to guide management of marine resources. For instance, Boldt and colleagues [39] summarized and synthesized the effects of climate and fishing on the Bering Sea and Gulf of Alaska ecosystem. Similarly, the ecological and economic consequences of hypoxia have been assessed in the Gulf of Mexico [40]. While these and similar efforts (reviewed by [41]) have applied portions of the IEA framework, the critical goal of integrating across multiple ocean-use sectors and numerous management objectives has not yet been fully achieved. However, a number of IEA efforts are occurring around the US. For instance, in Puget Sound a comprehensive scoping process [26] has led to an effort to identify ecosystem indicators and perform risk assessments and MSEs (Box 1). With a substantial budget and governance structure to implement management strategies emerging from the IEA [42], the lessons learned from the Puget Sound IEA will be instrumental in fine-tuning IEA efforts.

**Box 1. Puget Sound: An IEA Case Study**

The Puget Sound ecosystem includes 41,500 km^2^ of upland, freshwater, estuarine, and marine habitats, and is home to a large and increasing human population from Olympia, Washington north to Vancouver, British Columbia. Though renowned for its superficial beauty, substantial evidence exists that the processes supporting the Puget Sound ecosystem have been severely disrupted. In response, Washington Governor Christine Gregoire and the Washington legislature created the Puget Sound Partnership (PSP)—a public–private entity made up of citizens, governments, scientists, and businesses working to rehabilitate and conserve Puget Sound (http://www.psp.wa.gov/). By employing the IEA framework, the PSP and their partners developed an “action agenda” that promotes a healthy economy and thriving natural ecosystem. Application of the IEA steps in Puget Sound is outlined below.

**Scoping.** The PSP worked with their diverse members, a science working group, and the general public to operationalize the Governor's broad vision. An output of this initial scoping process included a definition of a healthy ecosystem as one that is resilient to changes, has built-in redundancy in its components, and has a representative sample of the diversity of species and habitats that characterized the historical state. The process is currently continuing with public engagement and stakeholder involvement. The goal is to articulate a plan with clear and measurable goals as well as management strategies that are consistent with achieving those goals.

**Indicator development.** A team of scientists solicited and organized expert judgment from the scientific community concerning potential ecosystem indicators for the region. Using a framework comparable to that proposed by Rice and Rochet [31], the team worked through proposed indicators and determined how well they meet criteria related to public awareness, cost-effectiveness, theoretical foundation, measurability, and the availability of historical data. Concurrent with this qualitative screening, researchers are using ecosystem models to rigorously evaluate indicator performance.

**Risk analysis.** Researchers in Puget Sound are following the conceptual approach developed by Smith and colleagues [19]. Susceptibility is being assessed by conducting a full inventory of the status and threats of key indicators, and has required developing a common set of definitions for categorizing threats. Simple spatial analyses of threats, by themselves, have proven useful in focusing attention on the regions of greatest concern. To define resiliency of community- or ecosystem-level indicators, ecosystem models (e.g., Ecopath with Ecosim; http://www.ecopath.org/) are being used to quantify how indicators respond to perturbations in the model. Pulse perturbations of varying intensity and on different ecosystem components are executed in the model, and time to recovery to the pre-disturbance state is used as a proxy for resilience.

**MSE.** While the initial steps of the IEA are in progress, researchers have started building an Atlantis ecosystem model of Puget Sound. Atlantis will serve as an operating model for formal MSE, using the Southeast Australian example described in the text [34] as a template. To provide management advice over the short-term, researchers are using conceptual models and statistical analyses to predict how key aspects of the ecosystem will change under different management portfolios.

**Monitoring.** Because there are diverse governmental, nongovernmental, and academic institutions in the Puget Sound region, a number of ongoing monitoring efforts exist in the region (e.g., http://wdfw.wa.gov/fish/psamp/). As results from other IEA steps come to the fore, these monitoring efforts may have to be altered or expanded to provide information on key indicators and management effectiveness.

## Concluding Thoughts

Historically, the cutting edge of ecosystem research was dominated by reductionist investigations [43]. Consequently, researchers and policy makers find themselves drowning in data while gasping for knowledge of how ecosystems respond to human activities [44]. While synthesis and integration are far more difficult to achieve than reduction [43], an understanding of the whole, not simply the parts, is clearly necessary to conserve and restore marine ecosystems and the services they deliver [45]. Masses of data simply cannot tell us how to implement EBM, or determine priorities for doing so. Likewise, simply tallying the status and trends of various components of the ecosystem cannot inform EBM. Instead, there is a clear need to actively integrate diverse physical, biological, and socioeconomic data and to think critically about the ways in which decisions affect tradeoffs among ecosystem goods and services valued by society. The IEA we describe here accomplishes this task and provides critical assessment support to the institutional framework supporting societal interests in healthy and productive ecosystems.

The time is ripe for a change in how marine resources are managed in the US [46]. Knowledge in the marine environment is immensely difficult to acquire, but over the decades marine scientists have steadily accumulated data, expertise, and tools. The future of marine ecosystems lies in the hands of policy makers, resource managers, scientists, and stakeholders who can take this collection of information, integrate it, and operationalize EBM. We have now reached a fork in the road between the well-trodden reductionist path and the less traveled synthetic way. IEAs, under the model we propose, point to a road less traveled, and we hold that this will make all the difference in defining a practical way forward in implementing EBM.

## Abbreviations

EBM: ecosystem-based management
IEA: integrated ecosystem assessment
MSE: management strategy evaluation
PSP: Puget Sound Partnership
